# A CONVERSATION ON TRANSLATION AND ADAPTATION OF EVIDENCE-BASED PROGRAMS FOR CAREGIVERS

**DOI:** 10.1093/geroni/igae098.0851

**Published:** 2024-12-31

**Authors:** Amy Eisenstein, Kenneth Hepburn

**Affiliations:** Chair; Discussant

## Abstract

There are currently a plethora of Evidence-Based programs designed to support caregivers. However, while caregivers represent a highly diverse population, cultural and linguistic characteristics and preferences are not always considered in the development of these programs. This symposium will bring together four research projects exploring lessons learned in their work around supporting culturally and ethnically diverse caregivers. Presenters will discuss specifics around when and why adaptations or new programs are needed and how to apply rigorous methods. The first paper will present lessons learned from the development and evaluation of a culturally tailored anxiety intervention for Latinx caregivers of older adults with cancer. The second paper will explore work on the translation and testing of an online program for Latinx family members who provide care for older people with dementia. Next we will hear about lessons learned from culturally adapting and tailoring an existing psychoeducation program for Black caregivers of persons with dementia. Finally, we will wrap up with a session describing a scoping review to identify common core components of family-centered interventions that could serve as a foundation for interventions that can be designed to support caregivers of all cultural and ethnic backgrounds. The session will be pulled together with a discussion on how we can take these lessons learned to continue to build programs in support of a culturally and ethnically rich population of caregivers, and will highlight gaps and further opportunities for research in the field.

